# Supplementary material for: Gene-expression-based T-Cell-to-Stroma Enrichment (TSE) score predicts response to immune checkpoint inhibitors in urothelial cancer
Source: Nat Commun. 2024 Feb 14;15:1349. doi: 10.1038/s41467-024-45714-0 (PMC10866910; doi:10.1038/s41467-024-45714-0)
Supplement: Supplementary file 1 — Supplementary Information [file 41467_2024_45714_MOESM1_ESM.pdf]

Supplementary Information

Gene-expression-based T-Cell-to-Stroma Enrichment (TSE) score predicts response to immune checkpoint inhibitors in urothelial cancer

Supplementary Figures

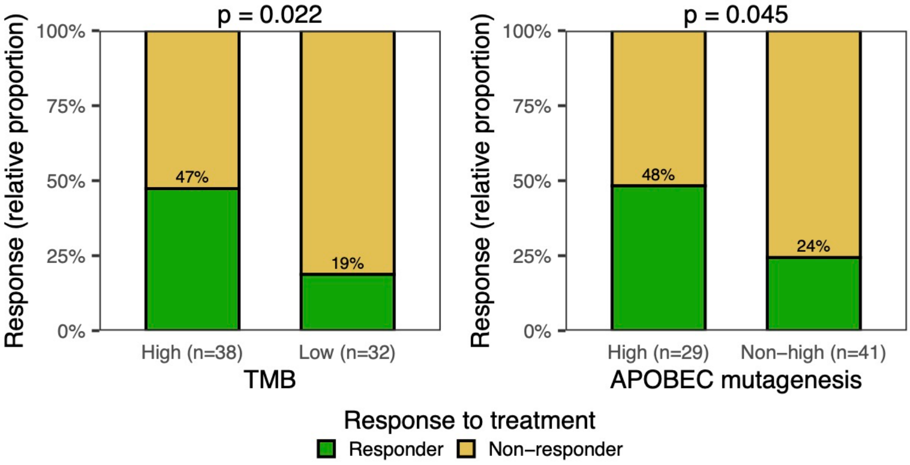

Supplementary Figure 1. TMB and APOBEC mutagenesis in responders and non-responders to pembrolizumab.
Bar graphs display the number of responders and non-responders in patients with high and low tumor mutational burden (TMB), and in patients with high and non-high (medium, low or no) APOBEC mutagenesis. P-values were determined using the two-sided Fisher's exact test.

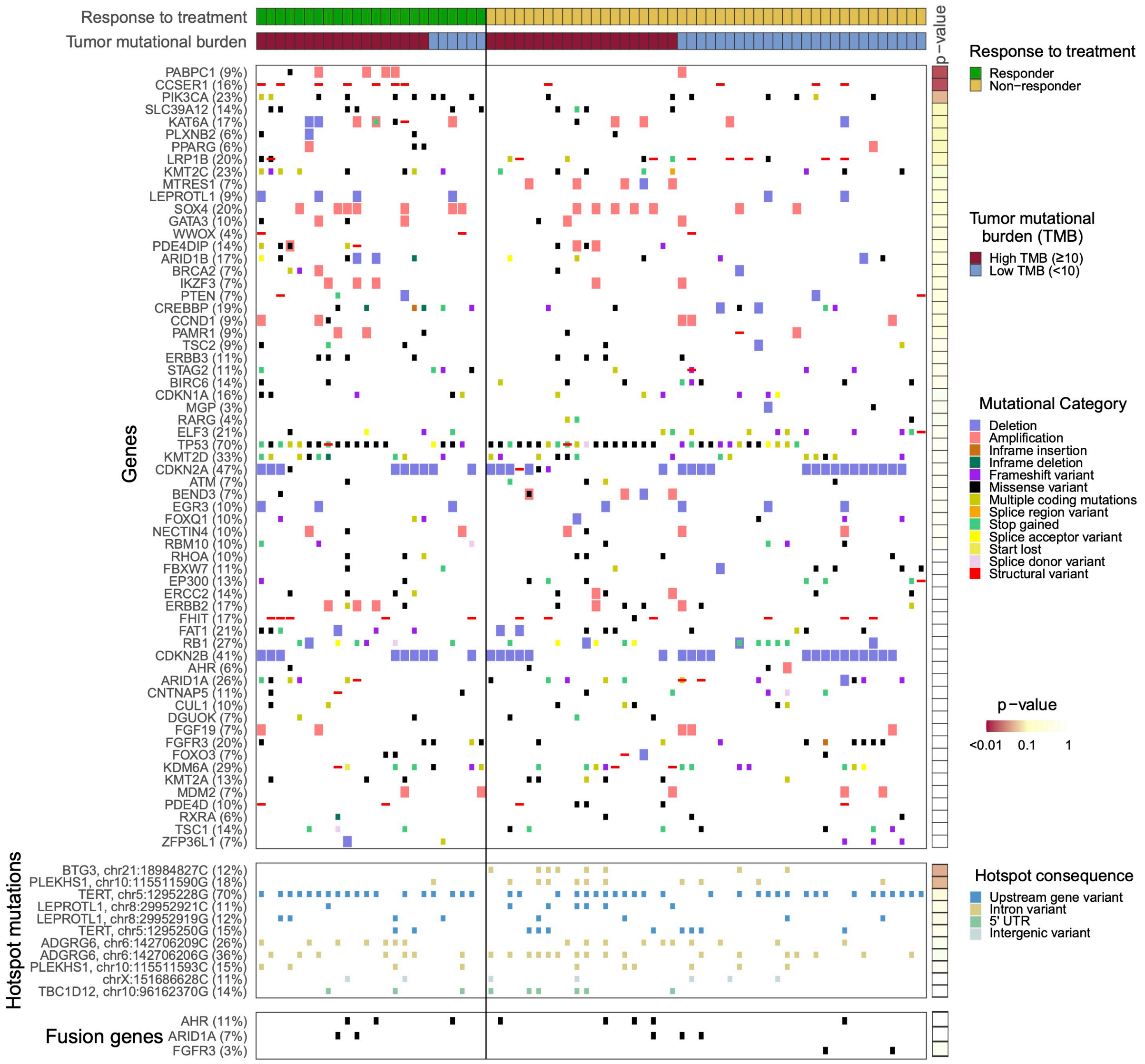

Supplementary Figure 2. Gene alterations in responders and non-responders to pembrolizumab. Overview of recurrent driver gene alterations, hotspot mutations and gene fusions in responders (n=24) and non-responders (n=46). Significantly mutated genes were estimated by dNdScv[1]; all genes with q<0.05 were considered driver genes. Recurrent focal copy number changes were estimated by GISTIC2[2]; genes in genomic regions with q<0.05 were considered significantly amplified/deleted. Gene fusions were detected from DNA using the pipeline described by the Hartwig Medical Foundation (https://github.com/hartwigmedical/). P-values of two-sided Fisher's exact test are shown on the right-hand side to reflect differential mutational status between responders and non-responders. After Benjamini-Hochberg correction, all p-values were not significant.

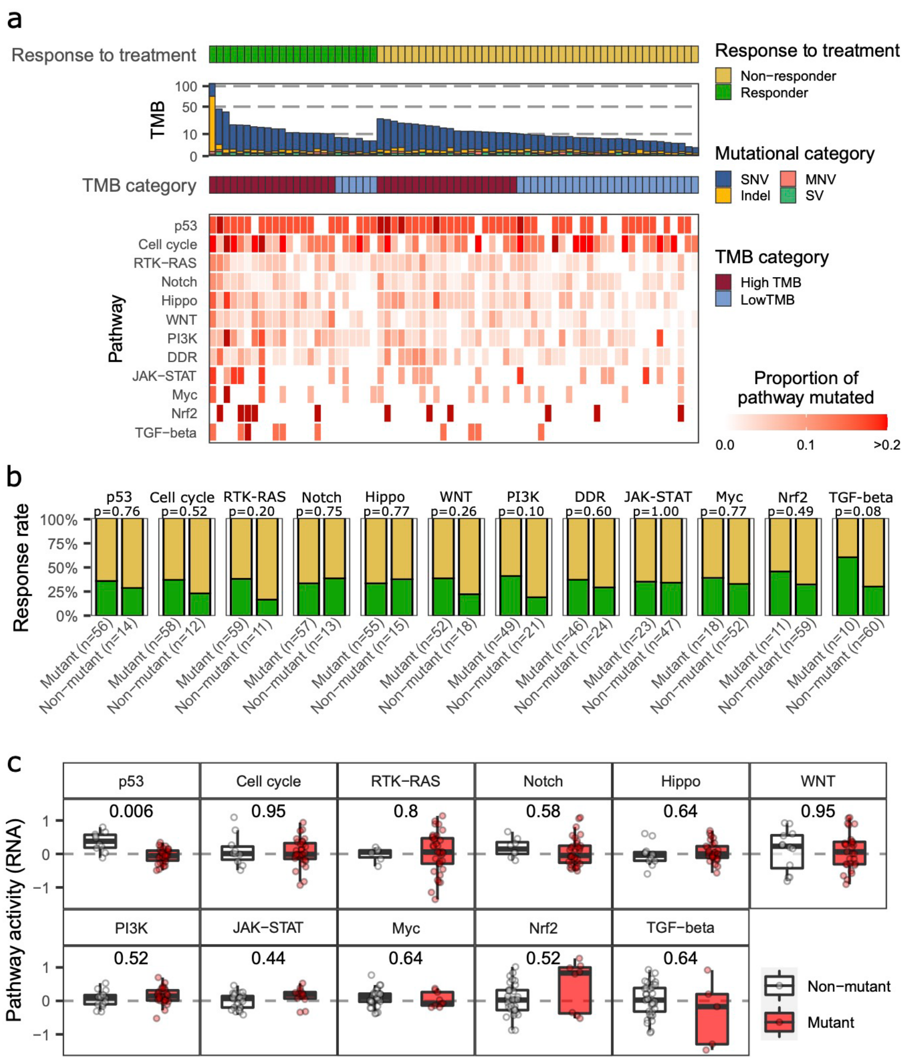

Supplementary Figure 3. Pathway alterations in responders and non-responders to pembrolizumab.

(a) The proportion of altered genes per pathway is visualized by a gradient of red color. Genomic alterations include: non-synonymous mutations, structural variants and deep copy number changes (as defined by GISTIC). Genes included in this analysis were previously published[3-5] and are listed in Supplementary Data 1. Patients are stratified according to treatment response (responders, n=24; non-responders, n=46), and according to tumor mutational burden (TMB). (**b**) Proportion of responders and non-responders in samples with and without genomic pathway alterations. Differences between responders and non-responders were assessed for statistical significance using the two-sided Fisher's exact test. No correction for multiple testing was applied. (**c**) Pathway activity at RNA level in patients with genomic alterations in at least one gene (mutant) *versus* patients without any genomic alteration in a specific pathway (non-mutant). Two-sided Wilcoxon-rank sum test with Benjamini-Hochberg correction was applied. Box plots show median, inter-quartile range (IQR: Q1-Q3) and whiskers (1.5xIQR from Q3 to the largest value within this range and 1.5xIQR from Q1 to the lowest value within this range).

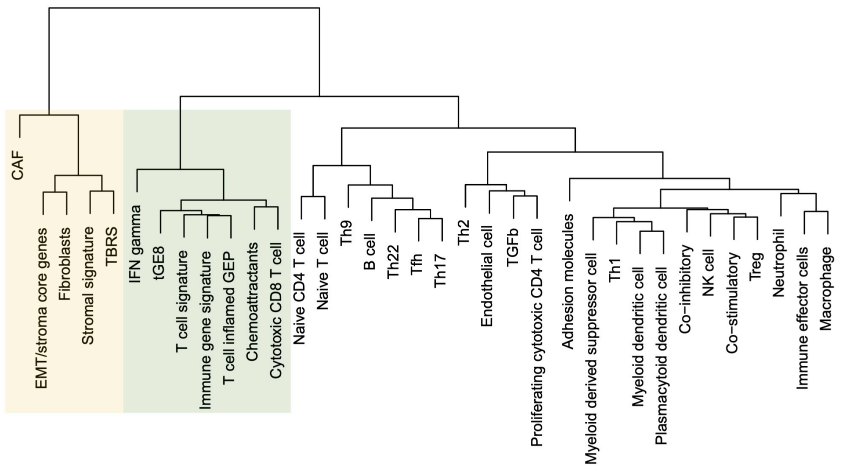

Category	Signature	SD of score
Stromal resident cells	CAF	1.31
T cells	IFN gamma	0.88
Stromal resident cells	EMT/stroma core genes	0.80
Stromal resident cells	Fibroblasts	0.79
T cells	Naive CD4 T cell	0.70
T cells	Naive T cell	0.69
T cells	tGE8	0.67
T cells	T cell signature	0.62
T cells	T cell inflamed GEP	0.62
T cells	Immune gene signature	0.61
T cells	Th9	0.60
Stromal resident cells	TBRS	0.54
T cells	Cytotoxic CD8 T cell	0.53
Stromal resident cells	Stromal signature	0.53
Immune cells (non T cell)	Macrophage	0.52
Immune cells (non T cell)	B cell	0.50
T cells	Chemoattractants	0.46
T cells	Immune effector cells	0.46
Immune cells (non T cell)	Myeloid derived suppressor cell	0.45
T cells	Tfh	0.43
T cells	Th17	0.43
Immune cells (non T cell)	Neutrophil	0.42
Immune cells (non T cell)	Plasmacytoid dendritic cell	0.38
T cells	Th22	0.37
Immune cells (non T cell)	Myeloid dendritic cell	0.37
T cells	Co-inhibitory	0.37
T cells	Th1	0.36
Immune cells (non T cell)	NK cell	0.36
T cells	Co-stimulatory	0.34
T cells	Adhesion molecules	0.34
Stromal resident cells	Endothelial cell	0.34
T cells	Treg	0.33
T cells	Th2	0.29
Stromal resident cells	TGFb	0.17
T cells	Proliferating cytotoxic CD4 T cell	0.12

Supplementary Figure 4. Selection of gene signatures for T cells and stromal resident cells and their products towards usage in the TSE score.
The dendrogram represents the hierarchical clustering of gene signatures. The two groups with the highest standard deviations (SD) across all samples are highlighted. The table on the right shows all signatures and their standard deviation.

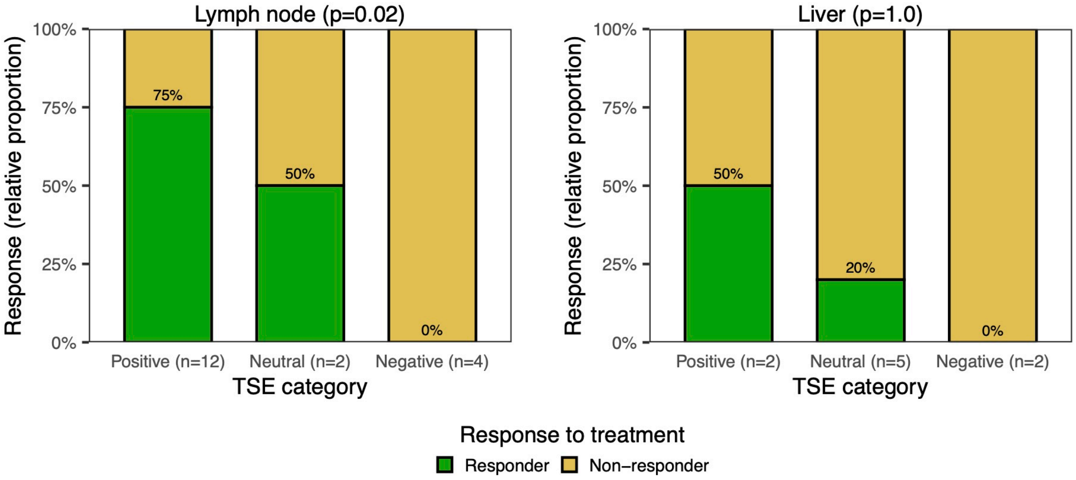

Supplementary Figure 5. The TSE score predicts response to pembrolizumab regardless of metastatic site.
Bar graphs display the relative proportion of responders and non-responders in patients with a positive, neutral, or negative TSE score according to metastatic site. P-values of TSE positive *vs* negative were determined using the two-sided Fisher's exact test. A significant p-value was achieved in samples derived from lymph nodes, while the number of samples from liver was too limited to achieve any statistical significance.

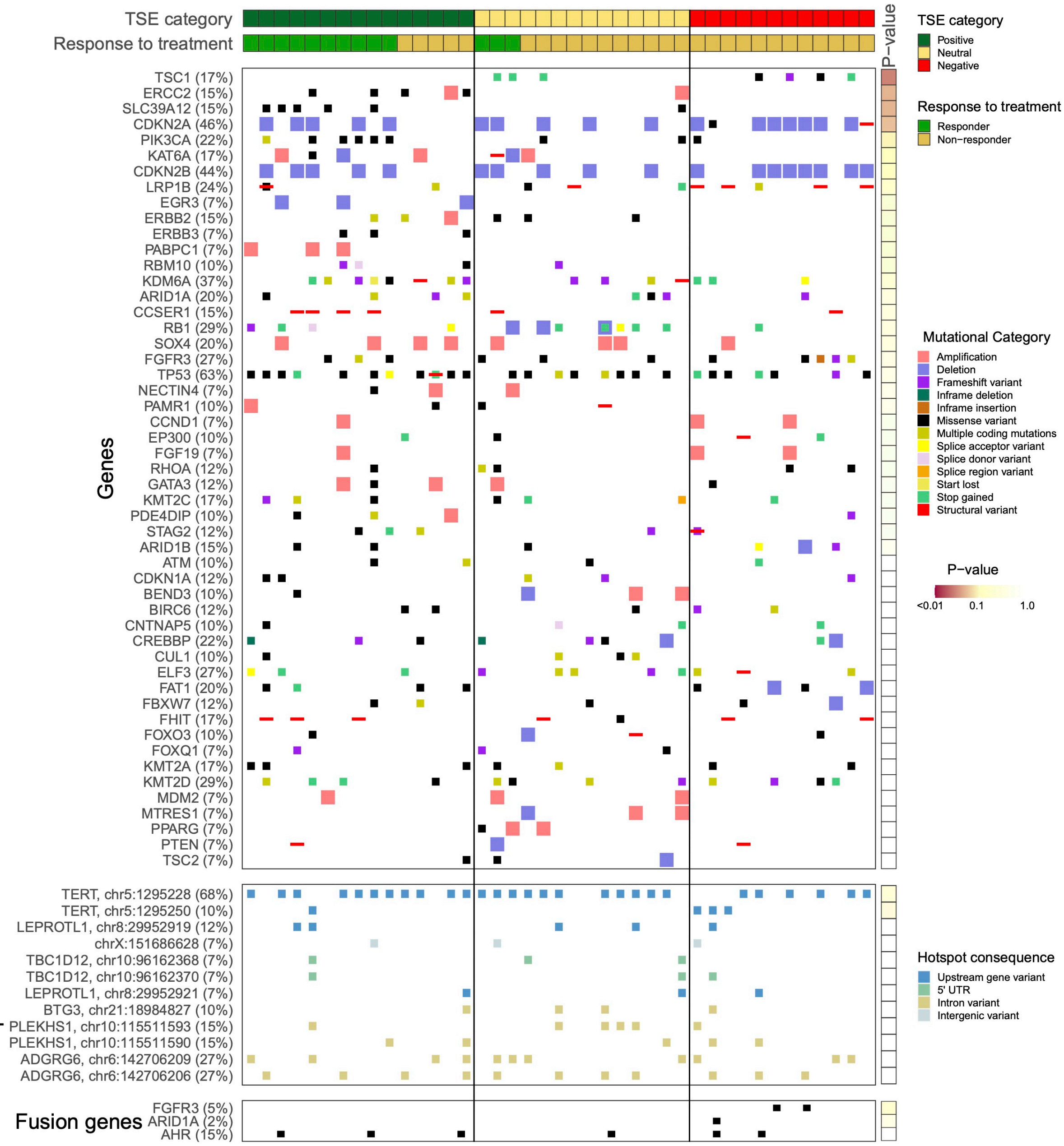

Supplementary Figure 6. Gene alterations according to TSE score.
Recurrent driver gene alterations, hotspot mutations and gene fusions are displayed according to the T cell-to-stroma enrichment (TSE) score (TSE positive, n=15; TSE neutral, n=14; TSE negative, n=12). See legend to Supplementary Figure 2 for details. P-values of two-sided Fisher's exact test are shown on the right-hand side to reflect differential mutational status between patients with a positive and negative TSE score. No significant difference was observed after applying Benjamini-Hochberg correction for multiple testing.

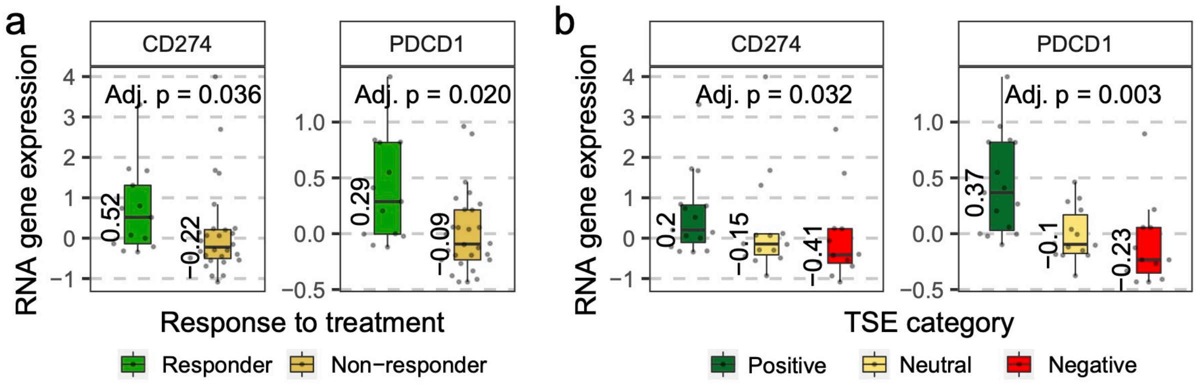

Supplementary Figure 7. PD-1 and PD-L1 gene expression according to response to pembrolizumab and TSE score.

Boxplots displaying *CD274* (PD-L1) and *PDCD1* (PD-1) gene expression according to **(a)** response to pembrolizumab (responders, n=13; non-responders, n=28), or **(b)** the T cell-to-stroma enrichment (TSE) score (TSE positive, n=15; TSE neutral, n=14; TSE negative, n=12). Differences between response *vs* non-response or TSE positive *vs* negative were tested for statistical significance using the two-sided Wilcoxon-rank sum test and Benjamini-Hochberg corrected. Box plots show median, inter-quartile range (IQR: Q1-Q3) and whiskers (1.5xIQR from Q3 to the largest value within this range and 1.5xIQR from Q1 to the lowest value within this range).

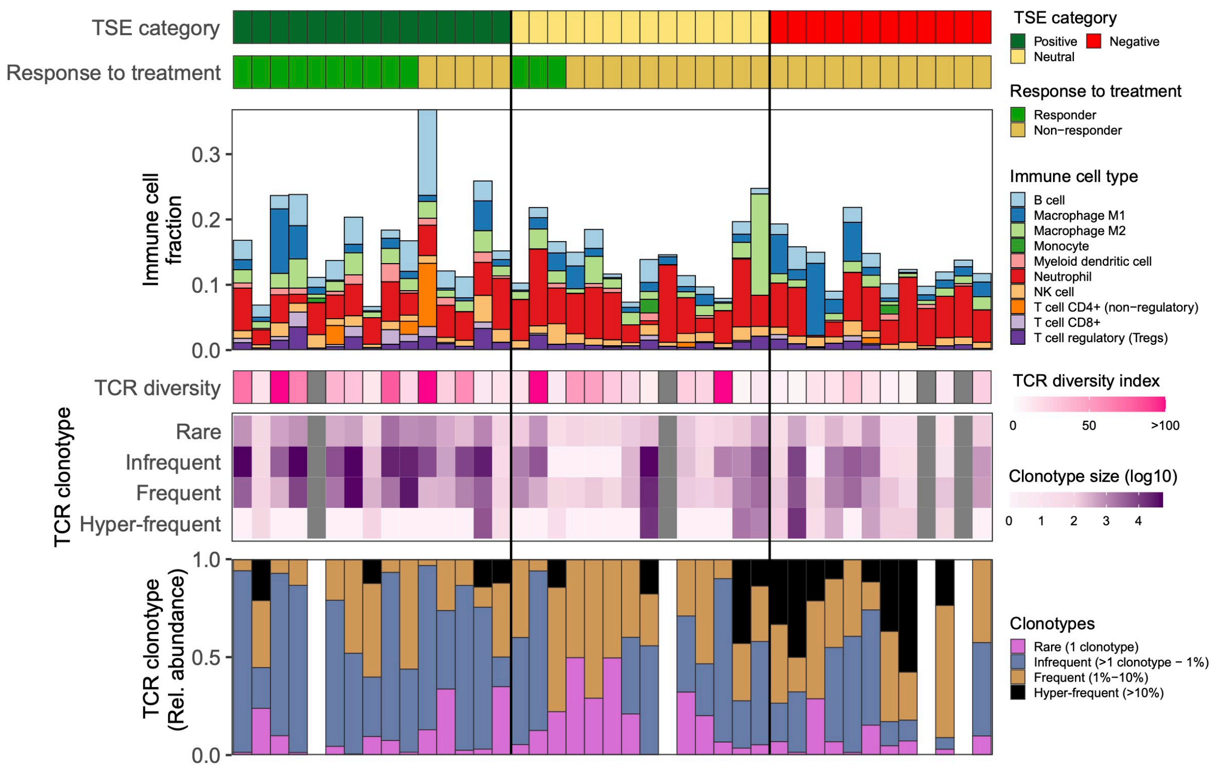

Supplementary Figure 8. Immune cell fractions and T cell receptor repertoire characteristics according to TSE score.

Immune cell fractions and T cell receptor (TCR) repertoire characteristics are displayed according to T cell-to-stroma enrichment (TSE) score (TSE positive, n=15; TSE neutral, n=14; TSE negative, n=12). Immune cell fractions were determined using quanTIseq[6,7]. TCR diversity, as well as size and relative abundance of TCR clonotypes were determined using MiXCR v3.0.13[18].

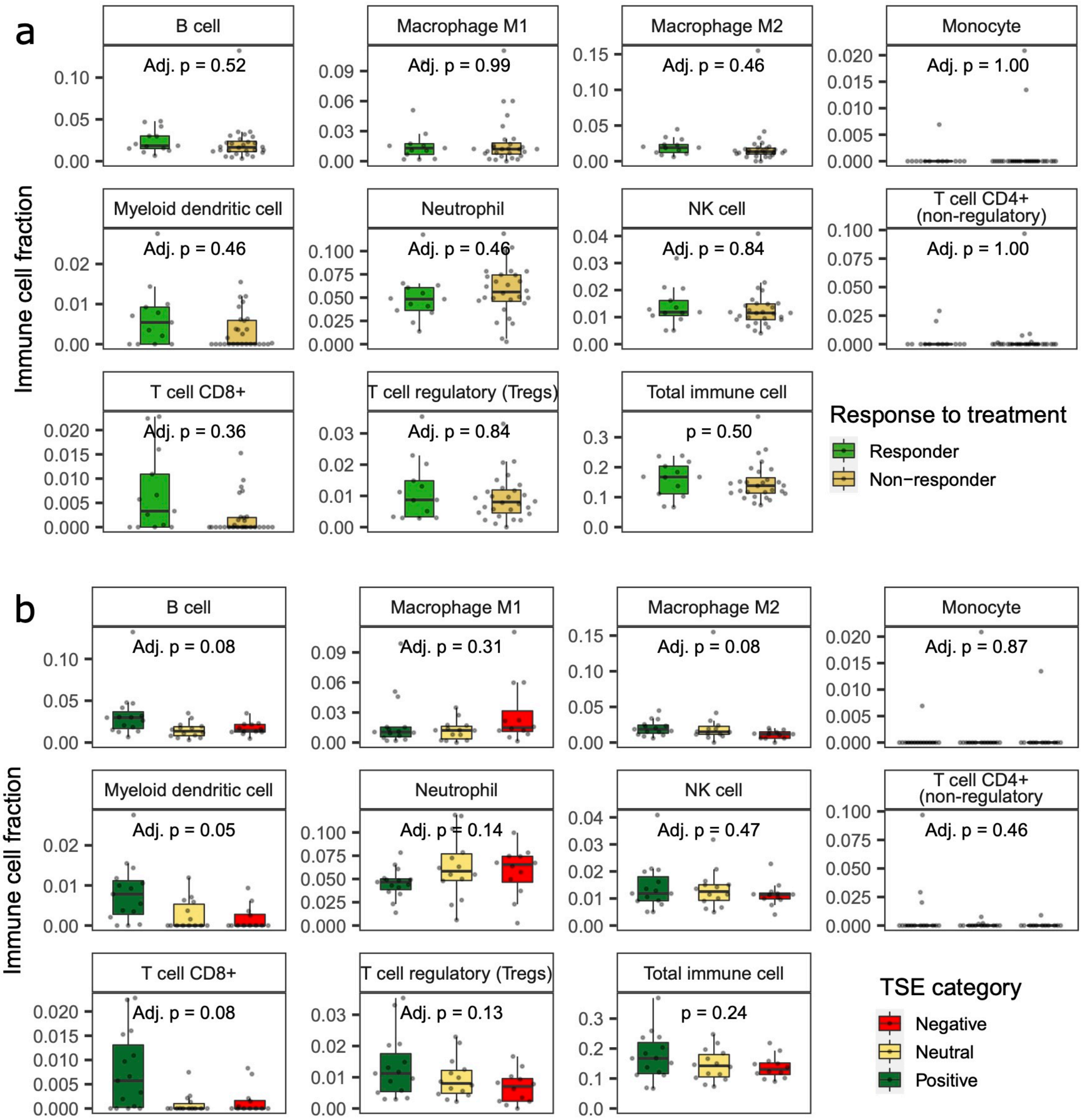

Supplementary Figure 9. Relative abundance of individual immune cell populations according to response to pembrolizumab and TSE score.

(a) Boxplots displaying individual immune cell fractions according to response to pembrolizumab (responders, n=13; non-responders, n=28). (**b**) Boxplots displaying individual immune cell fractions according T cell-to-stroma enrichment (TSE) score (TSE positive, n=15; TSE neutral, n=14; TSE negative, n=12). Immune cell fractions were determined using quanTIseq[14,15]. Differences between response *vs* non-response or TSE positive *vs* negative were assessed for statistical significance using the two-sided Wilcoxon-rank sum test and Benjamini-Hochberg corrected. Box plots show median, inter-quartile range (IQR: Q1-Q3) and whiskers (1.5xIQR from Q3 to the largest value within this range and 1.5xIQR from Q1 to the lowest value within this range).

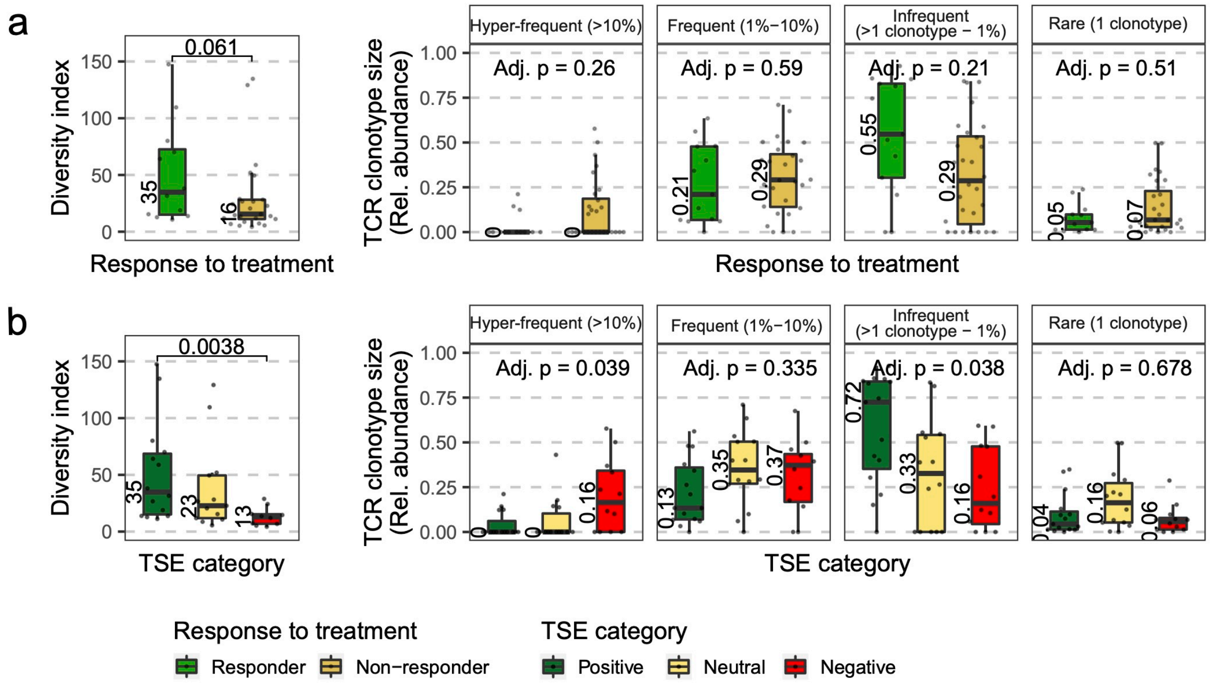

Supplementary Figure 10. T cell receptor repertoire clonality and diversity according to response to pembrolizumab and TSE score.

Boxplots displaying TCR diversity and relative abundance of TCR clonotypes according to **(a)** response to pembrolizumab (responders, n=13; non-responders, n=28), or **(b)** T cell-to-stroma enrichment (TSE) score (TSE positive, n=15; TSE neutral, n=14; TSE negative, n=12). TCR diversity and clonality were determined using MiXCR v3.0.13[16]. Differences between response *vs* non-response or TSE positive *vs* negative were assessed for statistical significance using the two-sided Wilcoxon-rank sum test and Benjamini-Hochberg corrected. Box plots show median, inter-quartile range (IQR: Q1-Q3) and whiskers (1.5xIQR from Q3 to the largest value within this range and 1.5xIQR from Q1 to the lowest value within this range).

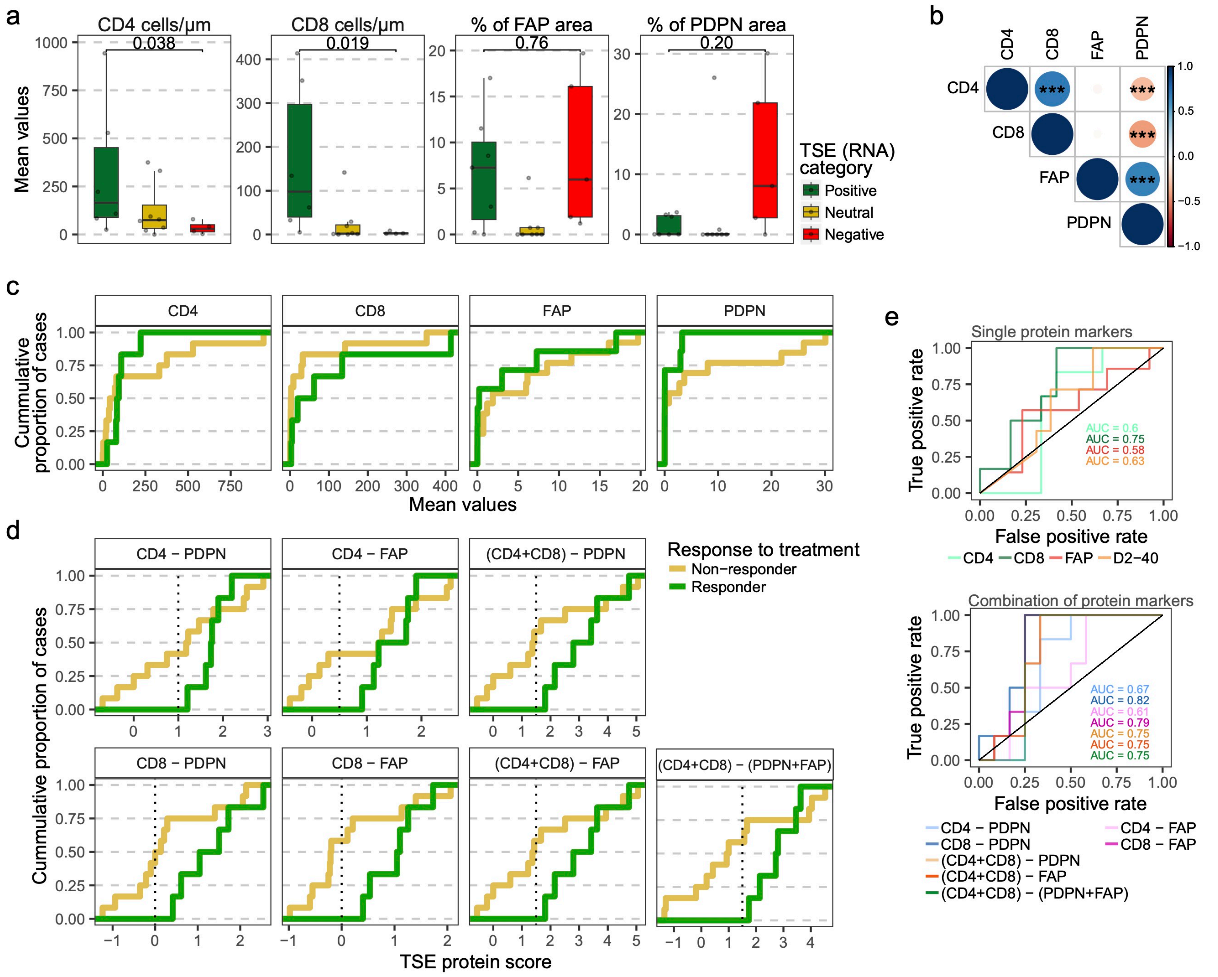

Supplementary Figure 11. Individual and combinations of protein markers according to response to pembrolizumab and TSE score.

(a) Boxplots displaying densities of CD4 and CD8 T cells and percentages of marker-positive areas of of stromal resident cells and their products (FAP and PDPN) in tumors with a positive (n=7), neutral (n=8), or negative (n=5) T cell-to-stroma enrichment (TSE) score. Differential expression of these markers between TSE positive *vs* TSE negative was compared using the two-sided Wilcoxon-rank sum test (without correction for multiple testing). Box plots show median, inter-quartile range (IQR: Q1-Q3) and whiskers (1.5xIQR from Q3 to the largest value within this range and 1.5xIQR from Q1 to the lowest value within this range). **(b)** Spearman correlation between protein markers (*** $p<0.001$). (**c**) Cumulative cases of responders and non-responders as a function of the individual protein marker values. (**d**) Performance of combinations of protein markers (TSE protein score) to select (~50% of) non-responders to pembrolizumab (indicated by a vertical dotted line). (**e**) Receiver operating characteristic (ROC) curves of individual protein markers and their combinations. The area under the curve (AUC) is displayed per condition.

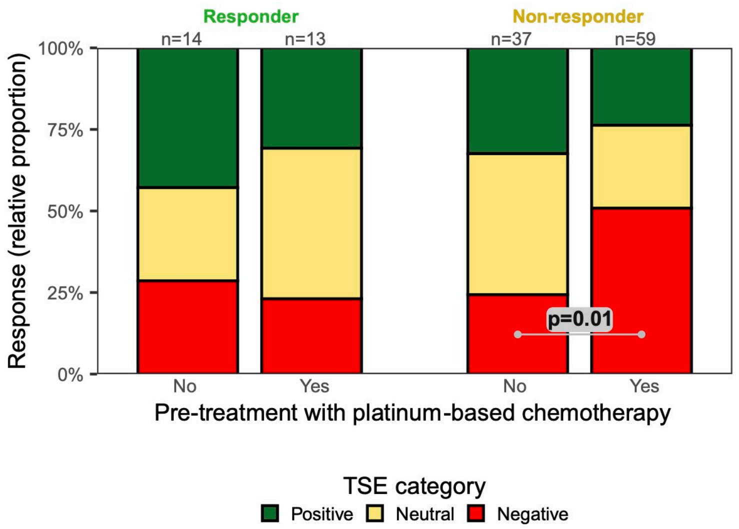

Supplementary Figure 12. Effect of pretreatment on the TSE score according to response to atezolizumab in the IMvigor210 cohort.

Bar graphs display the relative proportion of tumors with a positive, neutral, or negative TSE score in responders and non-responders. Patients in the IMvigor210 cohort who only received platinum-based chemotherapy (excluding BCG pre-treated), were considered pre-treated. Patients were considered treatment-naïve when neither intravesical BCG nor platinum-based chemotherapy was previously administered. The two-sided Fisher exact test was applied to compare the proportion of TSE negative patients between pre-treated *vs* treatment-naive among non-responders.

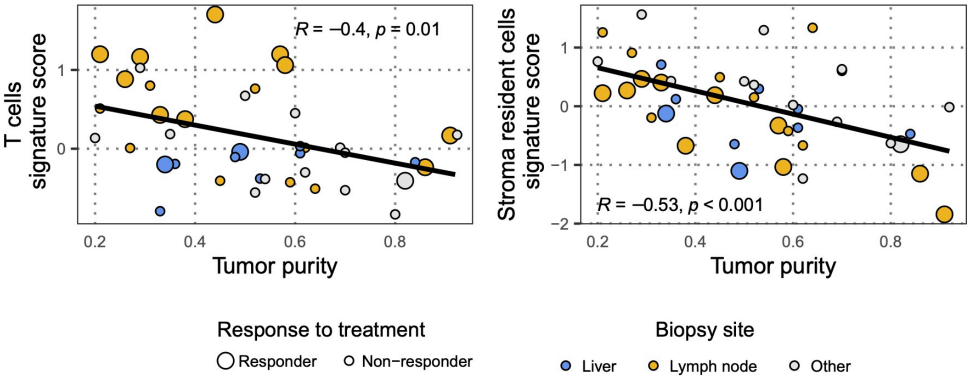

Supplementary Figure 13. Signature scores representing T cells and stromal resident cells and products negatively correlate with tumor purity.
Plots display Pearson correlations between signature scores that are used to estimate the TSE score and tumor purity according to Pearson (left: T cells; right: stromal resident cells and products). Details regarding gene expression signatures and determination of tumor purity are described in Materials and Methods.

Supplementary Tables

Supplementary Table 1. Characteristics of 70 patients with metastatic urothelial cancer who received treatment with pembrolizumab, and stratified by response to treatment at 6 months according to RECIST v1.1.

Cohort	Responder	Non-responder	Association with		
**n=70**	n=24	n=46	Response	OS	PFS
Age in years: median (range)	72 (42-85)	69 (48-83)	p=0.15	p=0.09[a]	p=0.09[a]
Male sex in numbers (%)	22 (92%)	36 (78%)	p=0.20	p=0.045	p=0.16
Primary tumor location					
Bladder	19 (79%)	35 (76%)	p=1.0	p=0.61[b]	p=0.79[b]
Upper tract	5 (21%)	10 (22%)	p=1.0		
Unknown	0	1 (2%)			
Systemic pretreatment					
Yes	18 (75%)	45 (98%)	p=0.005	p=0.026	p=0.005
Gemcitabine, cisplatin and/or carboplatin	16 (67%)	39 (85%)			
Methotrexate, vinblastine, doxorubicin, cisplatin	2 (8%)	4 (9%)			
Chemotherapy in combination with immunotherapy	0	1 (2%)[c]			
Other[d]	0	1 (2%)			
Radiotherapy pretreatment					
Yes	2 (8%)	15 (33%)	p=0.04	p=0.047	p=0.045
No	22 (92%)	31 (67%)			
Best overall response (RECIST v1.1)					
Complete response	3 (13%)	0		p= $1.1x10^{-11}$	p= $1.4x10^{-17}$
Partial response	18 (75%)	1 (2%)			
Stable disease	3 (13%)	7 (15%)			
Progressive disease	0	38 (83%)			
PD-L1 Combined positivity score					
Positive	5 (21%)	11 (24%)	p=1.0	p=0.31[b]	p=0.47[b]
Negative	8 (33%)	16 (35%)			
Unknown	11 (46%)	19 (41%)			
Biopsy sites					
Lymph node	14 (58%)	17 (37%)	p=0.13	p=0.14[f]	p=0.092[f]
Liver	3 (13%)	11 (24%)	p=0.35		
Soft tissue	3 (13%)	7 (15%)	p=1.0		
Other[e]	4 (17%)	11 (24%)			
RNA sequencing data available	13 (54%)	28 (61%)			
Immunofluorescence staining	7 (35%)	13 (65%)			

P-values for the association of response with categorical variables were estimated with the two-sided Fisher's exact test. For age, the two-sided Wilcoxon-rank sum test was applied. The log-rank test was used for comparing Kaplan-Meier overall survival (OS) and progression-free survival (PFS) curves.

[a]Age was dichotomized into young (age < median) and old.

[b]Unknown was excluded.

[c]Patient was randomized for nivolumab or placebo in trial.

[d]Other systemic pretreatments include a randomized trial treatment with adjuvant nivolumab or placebo treatment.

[e]Other biopsy sites include lung, bone, primary tumor, local recurrence, adrenal gland, and peritoneum (see also Fig 1).

[f]Others were excluded.

Supplementary Table 2. Gene expression signatures for T cells, other immune cells (non-T cells) and stromal resident cells and products.

**T cells and products (n=21)**	Individual signatures
Ayers et al.[9]	T cell inflammation gene expression profile (GEP), Immune gene signature, interferon (IFN) gamma
Hammerl et al.[10]	Co-inhibitory receptors, co-stimulatory receptors, chemo-attractants, immune effector cells, adhesion molecules
Jerby-Arnon et al.[11]	T helper type 1 cells (Th1), T helper type 2 cells (Th2), T helper type 9 cells (Th9), T helper type 17 cells (Th17), T helper type 22 cells (Th22), T follicular helper cell (Tfh), naïve CD4 T cells, regulatory T cells (Treg), cytotoxic CD8 T cells, naïve T cells
Mariathasan et al.[12]	Eight-gene cytotoxic T cell transcriptional signature (tGE8)
Oh et al.[13]	Proliferating cytotoxic CD4 T cells
Spranger et al.[14]	T cell signature
**Immune cells (non-T cells; n=7)**	
Jerby-Arnon et al.[11]	B cells, natural killer (NK) cells, myeloid derived suppressor cells, plasmacytoid dendritic cells, myeloid dendritic cells, neutrophil, macrophages
**Stromal resident cells and their products (n=7)**	
Hammerl et al.[10]	Fibroblasts, endothelial cells, transforming growth factor beta (TGFb)
Jerby-Arnon et al.[11]	Cancer associated fibroblasts (CAF)
Mariathasan et al.[12]	TGF-β-induced genes (pan-TGF-β responsive signature: TBRS)
Wang et al.[15]	EMT/stroma core genes
Yoshihara et al.[16]	Stromal signature

The complete list of genes per gene expression signature is available in Supplementary Data 3.

Supplementary Table 3. Area under the curve (AUC) of receiver operating characteristic curves for predicting response to pembrolizumab with gene expression signatures.

Category	Name	AUC	95% CI	Adj. p-value
Combined signatures				
	TSE score	0.88	0.78-0.99	
	T cells	0.75	0.58-0.92	
	Stromal cells	0.73	0.57-0.90	
	Other immune cells (non T cells)	0.62	0.40-0.84	
	T cells + Other immune cells	0.69	0.50-0.89	
Individual signatures				
T cells	IFN gamma	0.77	0.61-0.93	0.052
Stromal cells	TBRS	0.77	0.61-0.92	0.038
T cells	Cytotoxic CD8 T cells	0.76	0.60-0.92	0.049
T cells	tGE8	0.76	0.59-0.93	0.049
T cells	Th9	0.76	0.59-0.93	0.083
T cells	T cell inflamed GEP	0.76	0.59-0.92	0.052
Stromal cells	Fibroblasts	0.76	0.60-0.91	0.049
T cells	Th22	0.74	0.58-0.91	0.049
T cells	Naive CD4 T cells	0.74	0.55-0.93	0.067
T cells	Immune gene signature	0.73	0.55-0.91	0.040
T cells	Th2	0.73	0.54-0.91	0.074
Stromal cells	TGFb	0.73	0.57-0.88	0.035
Stromal cells	EMT/stroma core genes	0.72	0.56-0.88	0.038
T cells	T cell signature	0.72	0.53-0.90	0.040
T cells	Tfh	0.72	0.52-0.91	0.047
T cells	Chemoattractants	0.71	0.52-0.91	0.040
Stromal cells	CAF	0.71	0.53-0.88	0.035
T cells	Co-stimulatory receptors	0.69	0.48-0.89	0.040
Other immune cells	B cells	0.67	0.47-0.87	0.030
T cells	Co-inhibitory receptprs	0.67	0.48-0.86	0.031
T cells	Immune effector cells	0.66	0.47-0.86	0.031
T cells	Th17	0.66	0.45-0.87	0.038
T cells	Th1	0.65	0.45-0.85	0.030
Other immune cells	Plasmacytoid dendritic cells	0.64	0.43-0.85	0.030
T cells	Naive T cells	0.64	0.41-0.86	0.035
T cells	Proliferating cytotoxic CD4 T cells	0.63	0.42-0.84	0.030
Other immune cells	Myeloid derived suppressor cells	0.62	0.40-0.85	0.031
Other immune cells	Myeloid dendritic cells	0.62	0.41-0.84	0.030
Other immune cells	NK cells	0.62	0.42-0.82	0.030
Stromal cells	Stromal signature	0.62	0.41-0.83	0.030
Stromal cells	Endothelial cells	0.62	0.41-0.83	0.030
T cells	Treg	0.62	0.40-0.84	0.030
Other immune cells	Macrophages	0.60	0.39-0.81	0.030
T cells	Adhesion molecules	0.58	0.40-0.76	0.030
Other immune cells	Neutrophil	0.54	0.34-0.73	0.030

The receiver operating characteristic curves of individual signatures were compared against the TSE score applying the one-sided DeLong’s test. P-values were adjusted using the Benjamini-Hochberg method.

Supplementary References

1 Martincorena, I. *et al.* Universal Patterns of Selection in Cancer and Somatic Tissues. *Cell* **171**, 1029-1041.e1021 (2017).

2 Mermel, C. H. *et al.* GISTIC2.0 facilitates sensitive and confident localization of the targets of focal somatic copy-number alteration in human cancers. *Genome Biology* **12**, R41-R41 (2011).

3 Sanchez-Vega, F. *et al.* Oncogenic Signaling Pathways in The Cancer Genome Atlas. *Cell* **173**, 321-337.e310 (2018).

4 Leonard, W. J. Role of JAK kinases and stats in cytokine signal transduction. *Int. J. Hematol.* **73**, 271-277 (2001).

5 Taber, A. *et al.* Molecular correlates of cisplatin-based chemotherapy response in muscle invasive bladder cancer by integrated multi-omics analysis. *Nat. Commun.* **11**, 1-15 (2020).

6 Sturm, G. *et al.* Comprehensive evaluation of transcriptome-based cell-type quantification methods for immuno-oncology. *Bioinformatics* **35**, i436-i445 (2019).

7 Finotello, F. *et al.* Molecular and pharmacological modulators of the tumor immune contexture revealed by deconvolution of RNA-seq data. *Genome Med.* **11**, 34-34 (2019).

8 Bolotin, D. A. *et al.* MiXCR: Software for comprehensive adaptive immunity profiling. *Nat. Methods* **12**, 380-381 (2015).

9 Ayers, M. *et al.* IFN-γ-related mRNA profile predicts clinical response to PD-1 blockade. *J. Clin. Invest.* **127**, 2930-2940 (2017).

10 Hammerl, D. *et al.* Clonality, Antigen Recognition, and Suppression of $CD8^+$ T Cells Differentially Affect Prognosis of Breast Cancer Subtypes. *Clin. Cancer Res.* **26**, 505-517 (2020).

11 Jerby-Arnon, L. *et al.* A Cancer Cell Program Promotes T Cell Exclusion and Resistance to Checkpoint Blockade. *Cell* **175**, 984-997.e924 (2018).

12 Mariathasan, S. *et al.* TGFβ attenuates tumour response to PD-L1 blockade by contributing to exclusion of T cells. *Nature* **554**, 544-548 (2018).

13 Oh, D. Y. *et al.* Intratumoral CD4(+) T Cells Mediate Anti-tumor Cytotoxicity in Human Bladder Cancer. *Cell* **181**, 1612-1625.e1613 (2020).

14 Spranger, S., Bao, R. & Gajewski, T. F. Melanoma-intrinsic β-catenin signalling prevents anti-tumour immunity. *Nature* **523**, 231-235 (2015).

15 Wang, L. *et al.* EMT- and stroma-related gene expression and resistance to PD-1 blockade in urothelial cancer. *Nat. Commun.* **9**, 3503 (2018).

16 Yoshihara, K. *et al.* Inferring tumour purity and stromal and immune cell admixture from expression data. *Nat. Commun.* **4**, 2612 (2013).
