# Supplementary material for: Gene-expression-based T-Cell-to-Stroma Enrichment (TSE) score predicts response to immune checkpoint inhibitors in urothelial cancer
Source: Nat Commun. 2024 Feb 14;15:1349. doi: 10.1038/s41467-024-45714-0 (PMC10866910; doi:10.1038/s41467-024-45714-0)
Supplement: Supplementary file 3 — Description of Additional Supplementary Files [file 41467_2024_45714_MOESM3_ESM.pdf]

Description of Additional Supplementary Files

File Name: Supplementary Data 1
Description: List of gene core pathways.

File Name: Supplementary Data 2
Description: Master Table: Clinical and molecular variables.

File Name: Supplementary Data 3
Description: List of gene signatures representing T cells and stroma.
