# Supplementary material for: Gene-expression-based T-Cell-to-Stroma Enrichment (TSE) score predicts response to immune checkpoint inhibitors in urothelial cancer
Source: Nat Commun. 2024 Feb 14;15:1349. doi: 10.1038/s41467-024-45714-0 (PMC10866910; doi:10.1038/s41467-024-45714-0)
Supplement: Supplementary file 7 — Reporting Summary [file 41467_2024_45714_MOESM7_ESM.pdf]

nature portfolio

Corresponding author(s): Reno Debets

Last updated by author(s): Nov 8, 2023

Reporting Summary

Nature Portfolio wishes to improve the reproducibility of the work that we publish. This form provides structure for consistency and transparency in reporting. For further information on Nature Portfolio policies, see our Editorial Policies and the Editorial Policy Checklist.

Statistics

For all statistical analyses, confirm that the following items are present in the figure legend, table legend, main text, or Methods section.

n/a	Confirmed	
☐	☒	The exact sample size (*n*) for each experimental group/condition, given as a discrete number and unit of measurement
☒	☐	A statement on whether measurements were taken from distinct samples or whether the same sample was measured repeatedly
☐	☒	The statistical test(s) used AND whether they are one- or two-sided<br>*Only common tests should be described solely by name; describe more complex techniques in the Methods section.*
☒	☐	A description of all covariates tested
☐	☒	A description of any assumptions or corrections, such as tests of normality and adjustment for multiple comparisons
☐	☒	A full description of the statistical parameters including central tendency (e.g. means) or other basic estimates (e.g. regression coefficient) AND variation (e.g. standard deviation) or associated estimates of uncertainty (e.g. confidence intervals)
☐	☒	For null hypothesis testing, the test statistic (e.g. *F*, *t*, *r*) with confidence intervals, effect sizes, degrees of freedom and *P* value noted<br>*Give P values as exact values whenever suitable.*
☒	☐	For Bayesian analysis, information on the choice of priors and Markov chain Monte Carlo settings
☒	☐	For hierarchical and complex designs, identification of the appropriate level for tests and full reporting of outcomes
☐	☒	Estimates of effect sizes (e.g. Cohen's *d*, Pearson's *r*), indicating how they were calculated

Our web collection on statistics for biologists contains articles on many of the points above.

Software and code

Policy information about availability of computer code

Data collection	inForm® software v2.4.8, Qupath software v0.4.1
Data analysis	R software v4.1.0, R script TSEscore_ICIs v1.0.0, R2CPCT v0.4, PURPLE v2.49, MiXCR v3.0.13, DESeq2 v1.32.0, ReactomePA v1.44.0, Trimmomatic v0.39, STAR 2.7.6a, sambamba v0.7.0, FeatureCounts v1.6.3, RSEM v1.3.1, ConsensusClusterPlus v1.54.0

For manuscripts utilizing custom algorithms or software that are central to the research but not yet described in published literature, software must be made available to editors and reviewers. We strongly encourage code deposition in a community repository (e.g. GitHub). See the Nature Portfolio guidelines for submitting code & software for further information.

Data

Policy information about availability of data

All manuscripts must include a data availability statement. This statement should provide the following information, where applicable:

- Accession codes, unique identifiers, or web links for publicly available datasets
- A description of any restrictions on data availability
- For clinical datasets or third party data, please ensure that the statement adheres to our policy

Access to WGS and RNA-seq data were granted under request number DR-176 via the Hartwig Medical Foundation. Raw WGS and RNA-seq, and processed WGS data are freely available for academic use through standardized procedures at https://www.hartwigmedicalfoundation.nl. Clinical data with pseudonymized patient IDs are provided in the Source Data file. Processed data from the IMvigor210 cohort is publicly accessible without restriction. Data from the ABACUS cohort was

accessed by contacting directly the corresponding author of the study. Source Data for figures, including quantification of protein markers from tissue staining, are provided with this paper.

Research involving human participants, their data, or biological material

Policy information about studies with human participants or human data. See also policy information about sex, gender (identity/presentation), and sexual orientation and race, ethnicity and racism.

Reporting on sex and gender	Sex was part of the clinical data collection protocol. We report 83% of patients were male. Due to the limited number of samples, no systematic analysis was done to compare differences between sex.
Reporting on race, ethnicity, or other socially relevant groupings	This information was not part of the collection protocol and it is not reported.
Population characteristics	This is described in Supplementary Table 1.
Recruitment	Between March 1st 2013 and March 31st 2020, patients with advanced or mUC from 31 Dutch hospitals were included in the nationwide Center for Personalized Cancer Treatment (CPCT-02) biopsy protocol (NCT01855477). The study population consisted of 288 patients who were scheduled for 1st or 2nd line palliative systemic treatment. Fresh-frozen metastatic tumor biopsies and matched normal blood samples were collected from 256 patients as described previously29. WGS was successfully performed for 184 patients. Seventy patients started a new line of pembrolizumab monotherapy and were included in the current analysis. Matched RNA-seq was successfully performed on 41 patient-samples, and immunofluorescence stainings were performed for 20 of these patients. In 10% of cases, for first line chemotherapy-naïve patients, selection was based on a positive PD-L1 CPS, which may have introduced bias by selecting potential responders.
Ethics oversight	The study protocol was approved by the medical ethics review board of the University Medical Center Utrecht, the Netherlands. Written informed consent was obtained from all participants prior to inclusion in the trial.

Note that full information on the approval of the study protocol must also be provided in the manuscript.

Field-specific reporting

Please select the one below that is the best fit for your research. If you are not sure, read the appropriate sections before making your selection.

☒ Life sciences ☐ Behavioural & social sciences ☐ Ecological, evolutionary & environmental sciences

For a reference copy of the document with all sections, see nature.com/documents/nr-reporting-summary-flat.pdf

Life sciences study design

All studies must disclose on these points even when the disclosure is negative.

Sample size	The final sample size of 70 patients was the result of the inclusion criteria and not on statistical estimation. Similarly, the 41 patients included for RNA-seq was based on the success of the RNA-seq protocol. The sample size for immunohistochemistry, was based on the 41 samples with RNA-seq that showed the most contrasting TSE score values to increase the success of validation. Again, sample size was not based on any statistical test.
Data exclusions	Patients were excluded if no tumor biopsy was obtained, the biopsy was non-evaluable (tumor cell percentage <20%), or in case patients were not treated with pembrolizumab monotherapy after biopsy
Replication	Two independent cohorts were used for validation: the IMvigor210 (NCT02108652) and ABACUS (NCT02662309). We tried to have access to another cohort of mUC treated with pembrolizumab, but this was not possible due to timing (cohort has not been published yet).
Randomization	This was a retrospective study where all patients received the same treatment and randomization was not necessary
Blinding	This was a retrospective study where all patients received the same treatment and blinding was not necessary

Reporting for specific materials, systems and methods

We require information from authors about some types of materials, experimental systems and methods used in many studies. Here, indicate whether each material, system or method listed is relevant to your study. If you are not sure if a list item applies to your research, read the appropriate section before selecting a response.

Materials & experimental systems

n/a	Involved in the study	
☐	☒	Antibodies
☒	☐	Eukaryotic cell lines
☒	☐	Palaeontology and archaeology
☒	☐	Animals and other organisms
☐	☒	Clinical data
☒	☐	Dual use research of concern
☒	☐	Plants

Methods

n/a	Involved in the study	
☒	☐	ChIP-seq
☒	☐	Flow cytometry
☒	☐	MRI-based neuroimaging

Antibodies

Antibodies used

Monoclonal mouse anti-PD-L1 22C3 (PD-L1 IHC 22C3 pharmDx, Agilent Technologies, Carpinteria, CA, USA). Anti-CD3 and -CD8 (Akoya Biosciences, Marlborough, MA, USA), anti-FAP (EPR20021, Abcam) and anti-PDPN (Cell Marque).

Validation

The companion diagnostic assay of pembrolizumab (PD-L1 IHC 22C3 pharmDx) is a clinical-proven assay to select patients treated with pembrolizumab. Reference: Roach C, Zhang N, Corigliano E, et al. Development of a companion diagnostic PD-L1 immunohistochemistry assay for pembrolizumab therapy in non-small-cell lung cancer. Appl Immunohistochem Mol Morphol. 2016; 4:392–397.
The anti-CD3 and -CD8 (Akoya Biosciences, Marlborough, MA, USA) have been tested by several studies, the latest work citing other studies is: Jungen, SH, et al. Spatial distribution of CD3- and CD8-positive lymphocytes as pretest for POLE wild-type in molecular subgroups of endometrial carcinoma. 2023; 10: 1110529
The anti-FAP (EPR20021, Abcam). Validated in WB, IHC-P and tested in Human samples. It has been cited in 16 publications.
The anti-PDPN (Cell Marque) has been used extensively used to characterize the human cancer microenvironment. References: Lee, H.R., Roh, J., Gu, G.Y. et al. Differential expression of podoplanin in metastatic lymph node is associated with extranodal extension in oropharyngeal cancer. Sci Rep 12, 3665 (2022). https://doi.org/10.1038/s41598-022-07794-0; Wang, X. et al. Blocking podoplanin inhibits platelet activation and decreases cancer-associated venous thrombosis. Thromb Res 2021; 200:72-80.

Clinical data

Policy information about clinical studies

All manuscripts should comply with the ICMJE guidelines for publication of clinical research and a completed CONSORT checklist must be included with all submissions.

Clinical trial registration

NCT01855477

Study protocol

https://clinicaltrials.gov/ct2/show/NCT01855477

Data collection

Clinical data, including patient demographics, tumor characteristics, previous therapies and therapy outcome after study inclusion, were collected in electronic case report forms (Castor) and stored in a central database. Data collected with Castor is automatically stored on certified, compliant servers in the EU. This data is protected by, among others, two-factor authentication and complies with the applicable privacy (GDPR) laws.

Outcomes

The primary outcome of this study was to identify a new biomarker to predict response to immune checkpoint inhibitors in patients with metastatic bladder cancer and compare its predictive value with known biomarkers.
We achieved this outcome by a systematic analysis of WGS data, RNA-seq data and tissue straining of relevant markers. Two independent cohorts were used to validate the robustness of the new biomarker identified in this study.

Plants

Seed stocks

NA

Novel plant genotypes

NA

Authentication

NA
